# Head and Neck Imaging with a Dental CBCT Device: Comparison of 360° and 180° Rotation Angles in Effective Dose and Quantitative Image Quality in a Phantom Study

**DOI:** 10.2174/0115734056316438241203095215

**Published:** 2025-04-17

**Authors:** Hakan Amasya, Osman Günay, Fatih Kesmezacar, Duygu Tunçman Kayaokay, Nami Yeyin, Eylem Kekeç, Tülin Zengin, Kaan Orhan, Songül Çavdar Karaçam, Mustafa Demir

**Affiliations:** 1 Department of Oral and Dentomaxillofacial Radiology, Faculty of Dentistry, Istanbul University-Cerrahpaşa, Istanbul, Türkiye; 2 CAST (Cerrahpaşa Research, Simulation and Design Laboratory), Istanbul University-Cerrahpaşa, Istanbul, Türkiye; 3 Health Biotechnology Joint Research and Application Center of Excellence, Esenler, Istanbul, Türkiye; 4 Department of Biomedical Engineering, Faculty of Electrical and Electronics Engineering, Yıldız Technical University, Istanbul, Türkiye; 5 Medical Imag. Tech. Program, Vocational School of Health Services, Istanbul University-Cerrahpaşa, Istanbul, Türkiye; 6 Radiotherapy Program, Vocational School of Health Services, Istanbul University-Cerrahpaşa, Istanbul, Türkiye; 7 Department of Nuclear Medicine, Cerrahpaşa Faculty of Medicine, Istanbul University-Cerrahpaşa, Istanbul, Türkiye; 8 Nuclear Energy Research Institute, Secondary Standard Dosimetry Laboratory, Istanbul, Türkiye; 9 Department of Oral and Maxillofacial Radiology, Faculty of Dentistry, Ankara University, Ankara, Türkiye; 10 Ankara University Medical Design Application and Research Center (MEDITAM), Ankara, Türkiye; 11 Department of Dental and Maxillofacial Radiodiagnostics, Medical University of Lublin, Lublin, Poland

**Keywords:** Cone-Beam computed tomography, Radiation, Radiography, Dental, Contrast-to-noise ratio, Signal-to-noise ratio

## Abstract

**Objectives:**

This study aims to investigate the effect of full- and half-rotation angles on patient radiation dose and quantitative image quality in CBCT imaging of the head and neck region.

**Methods:**

A total of 67 TLDs were used for the dosimetry of 16 different regions in the head and neck slices of the anthropometric phantom. The Hyperion X9 Pro (MyRay, Cefla, Imola, Italy) CBCT device was used with a 90 kV pulsed beam and a 13x16e FOV size. Two separate imaging modes (Regular 360^ 0^ and Quick 180^ 0^) were tested, and the mA was determined by the software. Effective doses (EDs) were calculated using the coefficients recommended by ICRP 103 (2007). For the quantitative image quality tests, three VOIs were manually selected for three separate densities in image slices selected from the mandible, maxilla, and paranasal sinus regions of both volumes separately. Pixel values were averaged, and (SNR), contrast-to-noise ratio (CNR), and uniformity tests were conducted.

**Results:**

In 360^ 0^, ED was calculated as 1.903 mSv and the highest absorbed dose was found in the oral mucosa (1.566 mSv). In 180^ 0^, ED was calculated as 1.123 mSv and the highest absorbed dose was found in the right temporal squamous region (0.984 mSv). The reduction in ED was found to be 41% for full- and half-rotation angles. Quick/Regular ratios for SNR and CNR were changed between 0.83-0.91.

**Conclusion:**

The magnitude of reduction in ED was found to be higher than the quantitative image quality; however, the impact of this change on diagnosis should be analyzed according to the imaging purpose.

## 1 INTRODUCTION

Otto Walkhoff produced the first dental radiograph, beginning dental radiography in 1896 [1]. The limitations of planar imaging were addressed by computed tomography (CT) in the 1970s, however, alternative methods were required for dentistry due to high doses and expense. Cone-beam CT (CBCT) was introduced in 1998 and has since revolutionized dental imaging with applications ranging from implant planning and more [2-6]. In Turkey, dental imaging devices accounted for 38% of all radiation sources by 2021 (36% in 2020), comprising 39% panoramic, 4% CBCT, and 57% intraoral devices [7, 8]. By 2019, there were 203 different CBCT models worldwide, compared to just 23 in 2008 [2].

CBCT systems can be standalone or combined with panoramic and cephalometric imaging in 2-1 or 3-in-1 hybrid configurations. Current devices can be categorized into groups based on factors such as installation space size, the focal spot size, the voxel size, the kilovoltage range (kV), the milliamperage (mA) range, the beam type (pulsed or continuous), the dynamic range, the detector type, the rotation angle, the scan time, the exposure time, the weight, the cost, and other parameters. The Field of View (FOV) size options are widely available [2, 3]. Bornstein *et al*. categorized FOV sizes as small (≤ 40 cm^2^), medium (> 40 cm^2^ and ≤ 100 cm^2^), and large (> 100 cm^2^) based on the value representing the FOV surface (FOV height multiplied by diameter). Some devices offer an extended FOV size with the stitching technique based on sequential scanning of two neighboring areas and combining them from overlapping areas [2, 9].

In CBCT imaging, radiation dose levels are lower than in multi-slice CT (MSCT) but higher when compared with intraoral or panoramic imaging [2, 10-12]. In a meta-review of effective doses (EDs) in dental and maxillofacial CBCT, seven systematic reviews were analyzed using the ROBIS tool. The reported EDs were changed between 3.9 and 1073.0 µSv, categorized according to three heights of FOV and nine operating kV between 68 and 120. The authors emphasized the difficulty in comparing studies due parameters such as device technical specifications, measurement methods, and scanning protocol [13]. The dose span of ten different CBCT devices was compared, with the lowest and highest possible exposure parameters for each device, using the same methodology to record the radiation dose. The authors reported ED values between 17.2 µSv and 396 µSv, with average values of 31.6 µSv for the lowest and 209 µSv for the highest exposition parameters [14]. Another study calculated organ doses and EDs for a range of available protocols using a next-generation i-CAT dental CBCT scanner (Imaging Sciences International, Hatfield, PA) and reported that the high-resolution protocol required twice the dose required for regular resolution, and the dose was decreased by approximately 40% in the 180^ 0^ protocols when compared to the 360^ 0^ scanning [15]. The effect of FOV and rotation angle on dose in dental CBCT was investigated using a 3D Accuitomo 170 dental CBCT unit (J. Morita, Kyoto, Japan). In their study, the FOV sizes were changed between 4x4 cm^2^ and 17x12 cm^2^, and full-rotation (360°) and half-rotation (180°) protocols were included. Researchers reported 54 µSv and 303 µSv for full-rotation, and the average dose reduction was found to be 45% in half-rotation protocols [16]. A common aspect of studies on radiation dose in CBCT imaging is the need for data from different devices and parameters.

The EFOMP-ESTRO-IAEA protocol suggests several tests for evaluating quantitative image quality parameters in CBCT imaging. Uniformity is defined as the ability of a CBCT system to produce images of a homogeneous object with appropriate average pixel values, regardless of its position inside the object. Methods such as xyz-uniformity curves or Deutsches Institut für Normung (DIN, German Institute for Standards) can be used to evaluate uniformity noise [17]. Spatial resolution determines the size of the two smallest objects that can be distinguished, and methods such as modulation transfer function (MTF) can be used to evaluate the spatial resolution. Signal defines the pixel values that reflect the object correctly, while image noise refers to random variations in pixel intensity. The main reason for noise can be attributed to electronic, quantum, or structural factors, and excessive noise can mask the lesions or patient anatomy. The Signal-to-noise (SNR) ratio is defined as the ratio between the desired signal and the image noise. Low-contrast resolution is defined as the ability to distinguish a signal from the background and can be tested by calculating the Contrast-to-noise ratio (CNR) [17-19].

This study aims to measure the radiation doses received by critical organs in CBCT imaging of the head and neck region and compare the EDs in regular (360°) and quick (180°) imaging protocols. Moreover, to determine the absorbed dose, Thermoluminescent Dosimeters (TLDs) were placed in 16 body parts of the anthropometric phantom. The coefficients for ED calculation were selected based on the 2007 publication 103 from the International Commission for Radiological Protection (ICRP). The effect of two imaging protocols on image quality was compared with quantitative image quality tests such as SNR, CNR, or uniformity. Therefore, it is aimed at producing information that can be considered when deciding the radiographic parameters to improve the patient benefit/dose ratio.

## 2 MATERIALS AND METHODS

In this study, absorbed doses were calculated using an anthropometric phantom and a CBCT device with pre-calibrated TLDs. Additionally, EDs and digital image quality parameters were calculated from the produced data. CBCT volumes were acquired with full and half cycles, with other parameters kept constant, and the results were compared. This experiment was conducted without any human-related material, and approval from research ethics committees was not required.

### 2.1 Imaging Sample, Device and Protocols

The Alderson Radiation Therapy (ART) phantom (Radiology Support Devices, Long Beach, CA) representing an average adult female (155 cm, 50 kg) was used in this study. The average tissue density of the phantom is 0.985 g/cm^3^, and it is produced and marketed using suitable materials to imitate soft and hard tissues. The simulation of soft tissues shows an average density instead of the sharp differences found in the human body. The material is also cut away over the oronasal pharynx, trachea, and stem bronchi to make room for air. For hard tissues, highly detailed polymer moldings that reproduce the shape, mass density, and attenuation coefficients of cortical bone and spongiosa were used instead of real human bone. The manufacturer ensures that the bone properties of the product comply closely with the standards established by the International Commission on Radiation Units and Measurements (ICRU Report No. 44). The phantom is formed by 2.5 cm-thick sections transected horizontally, and slices are fixed with nylon rods between aluminum plates. A total of 16 dosimetry holes in the first 11 slices (Table **1**), which represent the head and neck region down to the sternoclavicular joint level, were used for placing dosimetry equipment, and unused holes were filled up using blank pins. The upper aluminum plate was not used to avoid the production of metallic image artifacts.

The Hyperion X9 Pro (MyRay, Cefla, Imola, Italy) CBCT machine with a tele-radiographic/cephalometric arm was operated with 230V and 50 Hz electrical input. The device had a CEI OPX/105-12 x-ray tube, a 0.5 mm focal spot (IEC 60336), 6.5 mm Al at 90 kV total filtration, and a 90 kV (< 5%) generator voltage in pulsed mode used for CBCT imaging. The anode material was made of tungsten (W), positioned at a 12° anode angle, and had a 30-kJ anode thermal capacity. Source-image receptor distance (SID) was 650 ± 5 mm, and the generator reference axis had a 5° tilt angle in lateral view. The CBCT detector was based on amorphous silicon technology with a CsI scintillator. The active surface was 162 x 162 mm (effectively 160 x 160), the spatial resolution was 3.94 lp/mm, and the pixel size was 127 x 127 µm. The image matrix size was 1280 x 1280 pixels, and the pixel depth was 16-bit (65,535 gray levels).

**Fig. (1):**
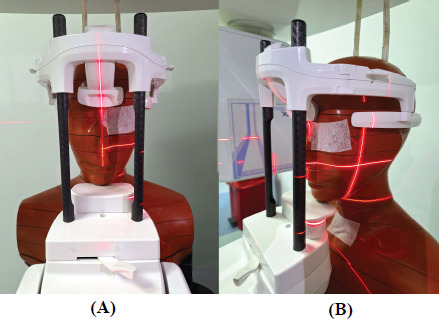
The phantom was fixed using six-point supports located on the chin, forehead, and temporals. Laser beams represent the FOV acquired in the first rotation for vertical stitching. (**A**): frontal view; (**B**): oblique view.

A 60-cm-high flat stand was used to support the phantom and access the device, and the device height was adjusted to maintain a neutral head position using a 2-speed motorized column. The phantom was positioned in a neutral spine position. Furthermore, due to the absence of an oral recess in the phantom, the bite stick could not be used, and the head was fixed with a total of 6 points. The position of the phantom was visually checked in each image acquisition by projecting a Class I (IEC 60825-1:2014) laser on the phantom's face (Fig. **1**).

The imaging protocols were selected as regular (360^ 0^) and quick (180^ 0^) modes, and two CBCT volumes were acquired. The FOV was fixed at the size of 13x16e, which requires double rotation for vertical stitching. Exposure parameters were modulated in real-time using Automatic Morphology Recognition Technology (MRT) during x-ray exposure in the range of 2–16 mA (< 10%) and 1–10.4 seconds (pulsed). The lateral and frontal scout images were obtained to check the spatial position of the phantom relative to the device, and minor corrections were performed if required. The volumetric data was acquired and reconstructed using an HP Z2 Tower G4 Workstation with an Intel® Xeon® E-2174G @ 3.8 GHz 4-core 8-thread CPU, 16 GB of RAM, 4 GB of GDDR5 AMD Radeon Pro WX3100 (10-bit) GPU, 1 TB of 7200 rpm SATA, and 500 GB of SSD HDD with the Windows 10 Pro for Workstations operating system and iRYS v15.0 software.

### 2.2 Dosimetry Protocols

The TLD material utilized for measurements was composed of chip-type MTS-100 (LiF:Mg,Ti). A total of 81 MTS-100 TLDs were pre-calibrated, and 67 TLDs were used to calculate the absorbed radiation dose in CBCT imaging. Caesium-137 (Cs-137) was used as a radioactive source (exposure: 50 cGy, hp(10)) only during the calibration session. The Harshaw 4500® TLD reader system and the WinRems® software were used for both the calibration and dosimetry sessions. Prior to any ionizing radiation exposure, TLDs were annealed at 400 °C for 1 hour, followed by 80 °C for 24 hours, and stored at room temperature for 24 hours. A further delay of 24 hours at room temperature was applied before the readings were completed once the radiation exposure was finished. For calibrations, the TLDs were irradiated using the Cs-137 radioactive source, and element correction coefficients (ECCs) were calculated with the Reader Calibration Factors (RCFs) using the reader system and the software sequentially. The calibration of the reader system was tested (exposure: 5 mSv) using 10 ‘calibration dosimeters’ in Calibrate Reader mode, and 69 TLDs were labeled as ‘field dosimeters’ to be used in dosimetry experiments. Among these 69 TLDs, a total of 67 TLDs were used in this present study: two pieces per anatomical region, 16 anatomical regions, two different imaging modes, and 3 pieces were engaged for background measurements. TLDs were prepared and transported using 3D-manufactured tools, as described in a previous study [18].

A total of 67 TLD read-out values were combined to obtain the mean absorbed dose in 16 anatomical regions (Table **1**). The average of the background dosimeter values was subtracted from the remaining TLD values, and the results of the TLDs in the same anatomical regions were merged by averaging both to calculate the absorbed dose. The EDs were calculated by multiplying the absorbed dose with the tissue weighting factors (Table **1**) and summing the products for each organ investigated. The tissue weight factors were adopted as suggested in ICRP Publication 103 in 2007 (Table **2**) [20].

### 2.3 Image Quality Tests

**Fig. (2):**
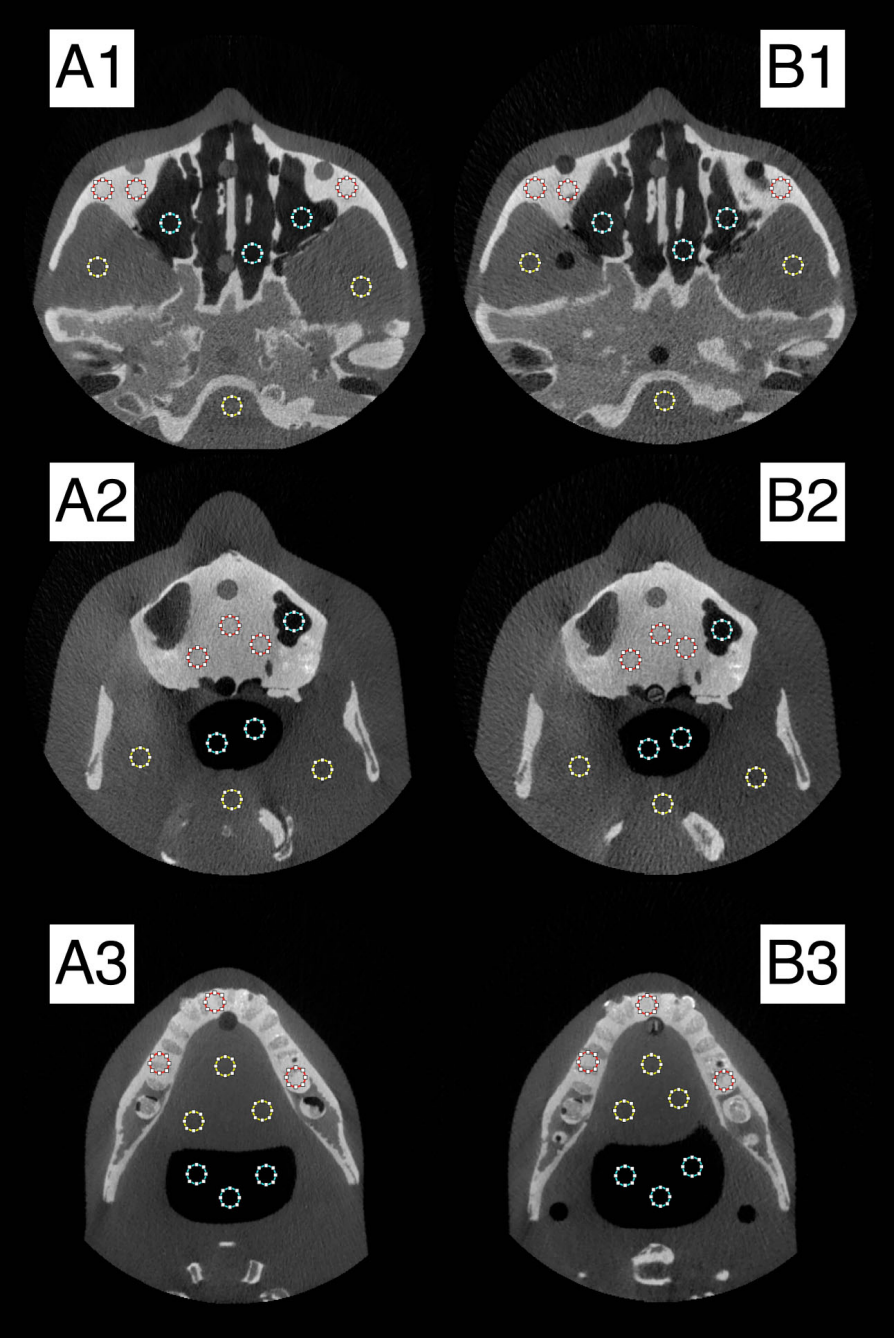
Axial slices were selected for image quality analysis, and 6 VOIs were determined in each image in an oval shape, making a total of 36. Letters represent two imaging parameters (**A**: Regular, 360^ 0^, **B**: Quick, 180^ 0^), while numbers represent vertical slice regions (1: paranasal sinus, 2: maxilla, 3: mandible). Bone, soft tissue, and air regions are demonstrated with red, yellow, and cyan colors, respectively.

The acquired volumetric data was exported in DICOM format to be used in quantitative image analysis tools. Phantom image samples were examined using Sante DICOM Viewer Lite (free, macOS, v1.0.1), and a total of six (three regular and three quick modes) axial image slices were selected in the mandible, maxilla, and paranasal sinus regions, as pairwise. Image samples (449x449 pixels, 16-bit) were imported to ImageJ separately, and in each image, three Volume of Interests (VOIs) representing bone, soft tissue, and air were adjusted separately using the circular VOI tool with a 6x6 diameter. A total of 54 VOIs (Fig. **2**) were determined on each slice. In addition, using the Measure function in the Analyze tab, the size of the selected area, average pixels value, stddev, min, and max values were calculated and saved in .csv format. The variables to be used in image quality equations were calculated by averaging the mean-pixel values of 3 VOIs in each variable (bone, soft tissue, and air, separately).

Formulas (1) and (2) were used to perform image qualification with signal-to-noise ratio (SNR) and contrast-to-noise ratio (CNR) parameters [18].

**Table:** 

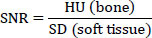	(1)

**Table:** 

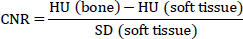	(2)

Here HU: Hounsfield Unit, SD: Standard deviation

For remaining image quality tests, suggestions in the EFOMP-ESTRO-IAEA protocol were modified to be used with the anthropometric phantom projections [17]. Uniformity was tested using both xyz uniformity curves (as average Houndfield Unit (HU) or mean gray values of the VOIs) and DIN methods. For calculating DIN, the mean pixel value of each VOI was obtained and averaged, and the sample with the maximum difference from the mean (Dmax) was determined and used with the formula below, where |P1-P2| is the contrast between the average pixel values of bone and the soft tissue entities as shown in formula (3).

**Table:** 

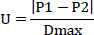	(3)

Voxel density values are represented in terms of mean and standard deviations in similar regions. The noise was evaluated using standard deviations in VOIs, and low-contrast resolution was calculated as CNR.

## 3 RESULTS

The tube current was determined to be 3 mA by the MRT feature, while the exposure times were 7.2 and 4.8 seconds for the Regular (360^ 0^) and Quick (180^ 0^) modes, respectively. The estimated radiation output decreased by an average of 34% among the two imaging modes (Table **3**).

For Regular (360^ 0^) mode, the lowest average absorbed radiation dose was found in the right thyroid (0.134 mSv), and the highest was found in the oral mucosa (1.566 mSv). The average absorbed dose for all dosimeters was 1.137 μSv. The ratio of right to left sides was found to be 1.6 for temporal squamous, 1.2 for parotid, and 0.9 for submandibular glands. In the Quick (180^ 0^) mode, the absorbed doses were found to be lowest in the thyroid (right) region (0.080 mSv) and highest in the right temporal squamous region (0.984 mSv). The average dose for all dosimeters was 0.689 mSv. The right-to-left side ratio was found to be 1.9 for temporal squamous, 1.2 for parotid, and 1 for submandibular glands. The average absorbed radiation dose was found to be 39.42% lower in Quick mode when compared with Regular mode. EDs were found to be 1.903 mSv for Regular mode and 1.123 mSv for Quick mode, and the reduction in ED was found to be 41% among the two imaging modes (Table **4**).

A total of 54 VOIs were used for the quantitative image quality tests. On each image slice, three VOIs were selected for each of the bone, soft tissue, and air entities separately. The area of a single VOI was calculated at 28.44. The minimum, maximum, mean, and standard deviation values of the pixel values in all VOIs are shown in Table **5**.

Bone, soft tissue, and air pixel values were obtained to be used in quantitative image quality tests by calculating the mean and standard deviations of the pixel values in the 3 VOIs for the same entity (Table **6**). The mean pixel values for bone were changed from 1239.2 to 2013.1 in regular mode and from 1247.7 to 1824.1 in quick mode. The mean pixel values for soft tissue were changed from -52.6 to 122.1 in Regular mode and from -92 to 102.3 in Quick mode.

The SNR, CNR, and DIN values are shown in Table **7**. The image quality ratio was found to be lower in quick mode when compared to regular mode in all tests and regions. The change in image quality in the paranasal region was found to be lower than in other regions, and a higher change was obtained in the DIN calculation for the mandibular region. The SNR ratio was calculated as 0.9, 0.84, and 0.83 in the paranasal sinus, maxilla, and mandible regions, respectively. In regular and quick modes, the highest test scores were obtained in the paranasal region, while the lowest were calculated in the maxilla region.

The diagram representing mean-pixel values and standard deviations in bone and soft-tissue VOIs is shown in Fig. (**3**). This diagram can be interpreted as xyz uniformity curves (mean-pixel value), voxel density (mean-pixel value and standard deviation), and noise (standard deviation in pixels) tests.

## 4 DISCUSSION

After the discovery of X-rays, the dental profession enthusiastically adopted them without fully considering the risks. Some pioneers raised awareness about radiation dangers, while others suffered amputations from exposure [21]. Modern innovations such as digital sensors and electronic timers have made radiography equipment less radiation-requiring over time, but the patient's dose still plays a crucial role in identifying the type and necessity of radiographic examination. ICRP 103 recommendations for tissue weighting factors were revised in 2007, and recent recommendations include salivary glands as individually weighted tissue and oral mucosa in the remainder of the tissues. The new list, updated according to the recommendations of the 1990s, draws attention to the reconsideration of ED calculations in the head and neck region [20].

The main imaging methods used in dentistry can be categorized as intraoral (periapical, bitewing, and occlusal) and extraoral (panoramic, cephalometric, and CBCT) techniques. Different techniques require varying amounts of radiation dose, and these values should be taken into account in the choice and clinical justification of radiographic examinations. Granlund *et al*. discussed the absorbed organ and EDs from intra-oral and panoramic imaging, using TLDs at 30 locations on the anthropometric head and neck. Intraoral radiographs were produced with 60 kV and 7 mA, 0.25-0.50 second exposure parameters. For maxillary and mandibular periapical imaging, a rectangular collimator was used to shine light on the photostimulable phosphor (PSP) plates. Panoramic images were obtained using three digital panoramic units with charge-coupled device-based and PSP-based detectors. EDs are calculated according to ICRP 103 recommendations, and the values for intraoral full-mouth (18 radiographs) examination (15 µSv) were reported to be lower than the average (36 µSv, range: 19-75 µSv) for panoramic radiography (for a single intraoral radiograph, average: 0.8 µSv, range: 0.1-2.6 µSv). Salivary glands and oral mucosa were reported to receive the highest organ dose in both imaging modalities [22]. Shousha *et al*. measured the EDs for a full-mouth intraoral examination, panoramic, and cephalometric imaging using TLDs in 12 anatomical sites of Rando-phantom. Estimated EDs were reported to be 8.5 µSv for full-mouth survey (14 radiographs), 2.2 µSv for panoramic, and 5.1 µSv for panoramic with cephalometric imaging (3 radiographs) [23]. In this study, EDs (1.123-1.903 mSv) were found to be higher than the previous studies, which investigated conventional planar dental radiography such as full-mouth intraoral projections or panoramic imaging. This finding is consistent with the statement that CBCT imaging requires a higher radiation dose than the conventional radiography techniques used in dentistry, based on the EDs previously reported. In Regular mode (360^ 0^), the highest absorbed organ doses were calculated in the oral mucosa (1.566 mSv), right parotis (1.493 mSv), and left lens (1.452 mSv), while in Quick mode (180^ 0^), the highest doses were obtained in the right temporal squamous (0.984 mSv), oral mucosa (0.927 mSv), and right parotis (0.916 mSv). Oral mucosa and salivary glands were found to be among the organs receiving the highest doses, similar to the study of Granlund *et al*., although the radiographic methods are different, such as planar and volumetric imaging [22].

Shatskiy calculated EDs from dental radiographic examinations based on the data obtained from forty-four intraoral, twelve panoramic, and seven CBCT units. The EDs for intraoral examinations were found to be in the range of 3.5–8.2 μSv for film-based and 1.2–2.5 μSv for digital detectors, and mean EDs were reported to be 22.9 μSv for panoramic and 530.6 μSv for CBCT imaging. The mean dose for CBCT examinations was reported to be over 200 times higher than the intraoral examinations with digital detectors and approximately 23 times higher than panoramic imaging [24]. ED values (1.123-1.903 mSv) obtained in this current study were found to be higher than in this study. Wrzesień and Olszewski conducted a study to determine the absorbed doses of the brain, spinal column, thyroid, and eye lens for panoramic, cephalometric, and CBCT imaging. The authors placed 18 TLDs in the anatomical points of an anthropometric Rando phantom and investigated 15 panoramic, 4 cephalometric, and 4 CBCT exposures. Mean absorbed doses were calculated to be higher in CBCT when compared to panoramic or cephalometric radiography and reported as 9.89±6.73 mGy, 0.85±0.55 mGy, and 0.4±0.3 mGy for the brain and spinal column, thyroid, and eye lens, respectively [25]. In the present study, absorbed doses for hypophysis (0.615–1.042 mGy) and spinal cord (0.648–1.22 mGy) were found to be lower, thyroid (0.080-0.134 mGy) was found to be comparable, and left lens (0.793–1.452 mGy) was found to be higher than the CBCT doses in the previous study.

The SEDENTEXCT Project reported an evidence-based guideline for CBCT in dental and maxillofacial imaging in 2012. The range of EDs for dento-alveolar and craniofacial type CBCT units was reported to be 11-674 and 30-1073 μSv, respectively. The ED range for intraoral (<1.5 μSv), panoramic (2.7-24.3 μSv), and cephalometric (<6 μSv) radiography was reported to be lower than the CBCT, while the dose range for MSCT maxilla-mandibular (280-1410) was relatively higher [26]. In this study, EDs were found to be comparable for Quick mode (1.123 mSv) and higher for Regular mode (1.903 mSv) than the dose range reported for the CBCT units.

Nardi *et al*. [6] compared head and neck EDs and quantitative assessments of the image quality of CBCT and MSCT in imaging of the head, cervical spine, ear, and dental arches. The authors placed 74 TLDs in the head and neck Alderson-Rando phantom and exposed the setup with 5 parameters for CBCT and 4 different scans in MSCT. CBCT examinations were conducted using a NewTom 5G CBCT (QR srl, Verona, Italy) device with the patient in a lying-down position (prone or supine). The ear and dental arches were recorded in high-resolution mode, while the head and cervical spine were recorded in standard mode. EDs for MSCT and CBCT were reported as 3490 and 248 μSv for the cervical spine, 1892 and 249 μSv for the head, 660 and 361 μSv for the ear, and 812 and 565-688 μSv for dental arches, respectively. The total absorbed dose of the remaining tissues was reported to be the highest among all organs in both head (5.9 mGy) and dental arches scans (20.4–21.7 mGy), followed by the lens of the eye (4.2 mGy) in the head and salivary glands (10.7–12.0 mGy) in dental arches scan modes. Absorbed doses for oral mucosa in CBCT imaging were reported as 2.4 mGy for the head and between 10 and 11.3 mGy for the dental arches. In this research, the effective doses (EDs) ranging from 0.739 to 1.252 were found to be higher when compared to the previous experiment. In the previous study, the CBCT device (NewTom 5G, SID: 970 mm) was operated with the patient in a lay-down position, while in the present study, the device (Hyperion X9 Pro, SID: 650 mm) was used with the patient in a standing position.. This parameter may be valuable when comparing calculated doses in both studies. In the previous study, image quality was evaluated by calculating MTF and CNR quantitatively. Overall, CBCT provided higher spatial resolution and lower contrast resolution when compared with MSCT, with a lower radiation dose. The CNRs for CBCT were reported to be between 5 and 25 in the previous study. In the current study, CNR values were found to be 9.97 and 11.7 in the maxilla, 24.62 and 28.99 in the mandible, and 39.31 and 43.43 in the paranasal sinus regions. Equipment-based parameters such as SID or exposure mode (low dose, regular, or high resolution) and ROI selection can be factors to consider when comparing the differences in both experiments.

Al-Okshi *et al*. conducted a systematic review to evaluate the ED of CBCT in imaging of the facial skeleton. The data were extracted from 38 studies, and it was emphasized that the lack of technical description of the devices, heterogeneity in measurement methods, and scanning protocols in the studies made it difficult to compare the EDs. The authors reported that EDs were in the range of 9.7–197 μSv for FOVs with a height ≤ 5 cm, 3.9–674 μSv for FOV heights between 5.1–10.0 cm, and 8.8–1073.0 μSv for FOVs ≥ 10 cm [13]. In the present study, the FOV size was fixed to 13x16e; however, the total height was achieved by vertical stitching of two adjacent shorter volumes acquired in two consecutive rotations. Therefore, when comparing the results of this research, it would be more appropriate to evaluate the dose calculations in our study as imaging twice with devices in the medium FOV group, rather than the large FOV group. da Silva Moura *et al*. in 2019 investigated the factors influencing the EDs in CBCT imaging in a systematic review. The authors included 44 articles and listed 13 factors, such as the size of the patient, region of interest, scan angle, exposure time, FOV size, kV, and mAs. The mAs was found to be the most evaluated factor, and the authors noticed that the rotation protocol was not evaluated sufficiently [27]. Rotation angle can be related to mAs, sample size, and image noise in CBCT [28].

Morant *et al*. conducted a study to investigate organs and EDs using an iCAT (Imaging Sciences International, Hatfield, PA) CBCT device with nine different FOVs, full- and half-rotation modes, and high-resolution acquisition. The ICRP reference computational models for adult males and females and Monte Carlo simulation were used, and EDs were reported between 25 and 66 μSv (DAP values between 191 and 556 mGy*cm^2^) for 360^ 0^, with approximately 40% lower doses in 180^ 0^. The authors also noted that the high-resolution protocol required twice the dose for standard resolution. The highest contributions to the ED calculation were reported as remainder tissues (31%, 27–36%), salivary glands (23%, 20–29%), thyroid (13%, 8–17%), red bone marrow (10%, 9–11%), and oesophagus (7%, 4–10%) [15]. The EDs calculated in this study (1.123–1.903 mSv) were found to be higher than in the previous study. In both studies, the oral mucosa and salivary glands were found to be the organs receiving the highest dose. Pauwels *et al*. conducted a study to evaluate the effect of FOV and angle of rotation on radiation dose in CBCT using a 3D Accuitomo 170 dental CBCT unit (J. Morita, Kyoto, Japan) and 148 TLDs. The authors reported EDs of 54 μSv for 4x4 and 303 μSv for 17x12 in 360^ 0^ rotation, and the use of 180^ 0^ rotation resulted in an average dose reduction of 45% [16]. In this study, the effect of regular (360^ 0^) and quick (180^ 0^) modes on the MyRay X9 Pro CBCT device was investigated in terms of patient dose and image quality. The largest FOV was selected (13x16e), which required double image acquisition for stitching, and mAs was determined as 14.4 and 21.6 for the Quick and Regular modes, respectively. EDs for Regular and Quick modes were found to be 1.903 and 1.123 mSv, respectively. According to the results of our research, the reduction of ED in the half-rotation protocol (41%) was found to be compatible with previous studies. The absorbed dose reduction was highest in the thyroid skin (62.9%), left submandibular gland (47%), and C1-C2 (Spinal Cord) (46.5%), while the lowest decrease was calculated in the ethmoid (anterior hypophysis) region (28.3%).

Rubens *et al*. emphasized that an asymmetric dose distribution can be observed in CBCT imaging [28]. Similarly, the ratio of right/left mean absorbed doses for temporal squamous, parotid, and submandibular glands in the current study was altered between 0.9 and 1.6 in regular mode (360^ 0^) and 1-1.9 in quick mode (180^ 0^) and showed an asymmetric distribution on both sides.

Goulston *et al*. conducted a systematic review questioning if altering operating potential (kV) and tube current exposure time product (mAs) on CBCT machines reduces radiation dose to patients undergoing dental and/or maxillofacial scans without a detrimental impact on image quality or diagnostic accuracy. A total of 22 studies regarding the effect of altering exposure parameters (current, kilovoltage, exposure time) on image quality were included, and 17 studies investigated how altering exposure parameters affect the image quality for specific diagnostic tasks. The authors concluded that by optimizing exposure parameters on most CBCT machines, it is possible to reduce patient doses while maintaining adequate diagnostic image quality. Previous studies evaluated image quality by calculating CNR, MTF, or other parameters using quality control phantoms [29]. In this study, image quality was evaluated by selecting VOIs on the selected slices of the anthropometric phantom.

SNR, CNR, and DIN ratios were changed in the range of 0.83–0.9, 0.85-0.91, and 0.81-0.92, respectively, according to the anatomical site. Quantitative image quality parameters were found to be lower in quick mode when compared to regular mode. A higher quick/regular ratio represents less change in image quality in terms of quantitative image quality evaluation. Quick and regular ratios in the paranasal region were found to be smaller when compared to the maxilla or mandible region. This may indicate that the quality loss in the paranasal sinus region, which has a relatively more homogeneous structure, is lower when compared to other regions. However, it should be noted that the image quality tests were conducted using the anthropometric phantom instead of an image quality control phantom, and the results are closely related to the selection of VOIs. Although VOI selection is limited in anthropometric phantoms, unlike homogeneous quality control phantoms, it can be considered more realistic in simulating biological tissues. The scope of quantitative image quality calculations in this study is to demonstrate the difference between full- and half-rotation as a ratio, and comparisons with different studies and devices are beyond the scope of our study.

Yeung *et al*. published a narrative review on the potential use of low-dose protocols for CBCT and reported that the low-dose protocols for CBCT imaging seem to have potential in various disciplines of dental medicine ranging from pediatric dentistry to oral and maxillofacial surgery. Dose reduction was reported to be usually achieved by mAs reduction, use of partial rotations, reduced number of projections, and larger voxel sizes, but seldom by kV reduction [30]. Kaaber *et al*. conducted a systematic review of low-dose CBCT protocols in three stages of implant therapy (planning, insertion, and follow-up examination of peri-implantitis), and reduction of the exposure parameters of kV, mA, resolution (through increased voxel size), exposure time, and scanning trajectory were reported as the low-dose protocols. The authors concluded that the use of low-dose CBCT protocols does not impact objective image quality assessment in any stage of implant therapy [31]. van Bunningen *et al*. investigated the differences in orthodontic diagnostic measurements on lateral cephalograms synthesized from ultra-low dose-low dose (ULD-LD) CBCT scans and reported that the variations in cephalometric measurements were small and suggested that this method should be considered for orthodontic purposes [32]. However, the presence of metallic restorations in the mouth (such as fillings, implants, crowns, bridges, *etc*.) increases the likelihood of beam hardening artifacts, and this is affected by the dose protocol administered. Codari *et al*. compared the influence of different CBCT devices, high-density materials, and field of views (FOVs) on the presence of metal artifacts, using three customized acrylic resin phantoms containing high-density materials cylinders made of titanium, copper–aluminum alloy, and amalgam. The authors reported variances of up to 67% in volume measurements and emphasized the importance of adjusting the parameters with a case-based approach [33].

As a general approach, it may be advisable to opt for a low-dose protocol in cases requiring a major geometric examination, such as the rough determination of the position of impacted teeth in orthodontics. Moreover, low-dose protocols are preferable in younger and slimmer patients. Indeed, a low dose also leads to a loss in image quality. This may, for example, limit the detection of possible root resorption in other teeth in the area when examining an impacted tooth. Furthermore, in cases where there are dense metallic restorations in the mouth, a high-dose protocol may be considered, not to reduce the dose but to reduce artifacts. In choosing the appropriate dose protocol, patient-specific evaluation is required, and it is essential to select the minimum dose without compromising diagnostic goals. According to the results of our study, when comparing half and full rotation, the percentage gain in patient dose is greater than the loss in image quality. However, it should be kept in mind that the low-dose protocol may be insufficient in issues such as medical design and surgical guide planning. Nevertheless, research is ongoing on software compensation and improvement of situations that lead to loss of image quality, such as the half-turn protocol or metal artifacts [34-36].

In this study, irradiation was performed with a single CBCT device, which can be considered a limitation. In particular, studies comparing different devices using similar methods are useful in showing the variation between products on the market. At the same time, our study focused only on comparing the volumetric image acquisitions made with the low-dose and routine protocols and did not aim to compare different devices. Another limitation of our study is that both irradiations were made using a single FOV size. CBCT devices offer various FOV sizes, both relatively larger and smaller, and organ doses vary depending on the anatomical region that small FOV fields target. Our study investigated two protocols in a fixed FOV, which required vertical stitching. Evaluating the effect of smaller FOV sizes in different anatomical regions may be the subject of future studies. In this study, slices obtained with an anthropometric phantom were used for image quality tests. This is a limitation compared to the quality control phantom, nevertheless, the anthropometric phantom may be considered superior in reflecting the real patient. However, the study could be improved by using phantoms of different sizes and genders. It is, however, important to note that all these phantom types are limited in reflecting complex situations in the oral cavity, such as metallic restorations and missing teeth.

## CONCLUSION

According to the results of our study, by reducing the rotation angle from 360° to 180°, the ED in CBCT imaging of the head and neck region decreased by 41%. Quantitative image quality ratios were calculated between 0.81-0.92. The change in image quality in the paranasal region was found to be less compared to other regions. The results of this study may contribute to the decision-making process and optimization of dental radiographic examinations. Percentage-wise, the gain in dose is greater than the loss in image quality. Nevertheless, risking adequate diagnostic information in the pursuit of dose gain may be pointless in terms of acquisition repetitions. The results of this study support the suggestion that a low-dose protocol can be chosen in cases where major anatomical structures are to be evaluated and do not require small details, while in cases such as the presence of metal restorations in the mouth, the low-dose protocol may be avoided.

## Figures and Tables

**Fig. (3):**
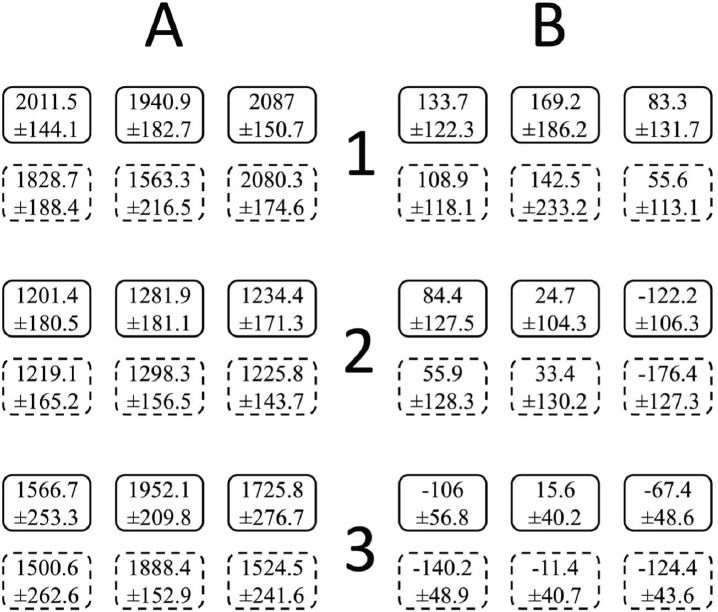
Diagram for xyz uniformity curves (mean-pixel value), voxel density (mean-pixel value and standard deviation), and noise (standard deviation in pixels) tests. Each box contains the mean and standard deviation of pixel values in the relevant bone and soft-tissue VOIs. Solid edges represent Regular (360^ 0^), while dashed edges represent Quick (180^ 0^) mode. (**A**): bone; (**B**): soft tissue; 1: paranasal sinus; 2: maxilla; 3: mandible.

**Table 1:** Distribution of the TLD locations in phantom slices, corresponding organs, and the recommended tissue weighting factors in ICRP 103 (2007) [20].

**Phantom ** **Slice**	**Organ**	**ICRP 103 Factor (2007)**
3	Hypophysis	0.04
3	Right temporal squamous	0.04
3	Left temporal squamous	0.04
3	Ethmoid (anterior hypophysis)	0.04
3	Right orbita	-
3	Right ossification chain	0.04
5	Oral mucosa (Upper tongue, Hard palate)	0.72
6	C1-C2 (Spinal cord)	0.16
6	Right parotid	0.04
6	Left parotid	0.04
7	Sublingual gland	0.04
6	Right submandibular gland	0.04
6	Left submandibular gland	0.04
10	Thyroid (right)	0.16
3-skin	Left lens	0.04
10-skin	Thyroid skin	0.04

**Table 2:** Tissue weighting factors suggested in ICRP 103 (2007) [20].

**Tissue**	**Tissue Weighting Factor wT**	**ΣwT**
Bone-marrow (red), colon, lung, stomach, breast, remaining tissues*	0.12	0.72
Gonads	0.08	0.08
Bladder, oesophagus, liver, thyroid	0.04	0.16
Bone surface, brain, salivary glands, skin	0.01	0.04
Total		1.00

Note: * Remaining tissues: Adrenals, extrathoracic region, gall bladder, heart, kidneys, lymphatic nodes, muscle, oral mucosa, pancreas, prostate (♂), small intestine, spleen, thymus, uterus/cervix (♀).

**Table 3:** Exposure parameters were modulated using Automatic Morphology Recognition (MRT) technology by analyzing the phantom anatomy in real-time.

**Mode**	**Exposure** **Time**	**mAs**	**Air Kerma** **(mGy)**	**DAP** **(mGy.cm^2)**	**DLP** **(mGy.cm)**	**CthIw** **(mGy)**	**CthIvol** **(mGy)**
Regular (360^ 0^)	7.2	21.6	4.41	395.83	18.27	1.02	1.15
Quick (180^ 0^)	4.8	14.4	2.94	263.89	12.07	0.67	0.76

**Table 4:** Average radiation doses absorbed by the organs in mSv units and percentage of change in Regular (360^ 0^) and Quick (180^ 0^) modes.

**Organ**	**Mode**	**Minimum (mSv)**	**Maximum (mSv)**	**Mean**±**SD (mSv)**	**Dose Reduction (%)**
Hypophysis	Regular*	0.956	1.128	1.042±0.122	40.93
	Quick**	0.557	0.673	0.615±0.082	
Right temporal squamous	Regular*	1.332	1.477	1.404±0.103	29.94
	Quick**	0.919	1.049	0.984±0.092	
Left temporal squamous	Regular*	0.89	0.898	0.006±0.006	41.87
	Quick**	0.51	0.529	0.52±0.014	
Ethmoid (anterior hypophysis)	Regular*	0.942	1.105	1.023±0.115	28.25
	Quick**	0.676	0.792	0.734±0.082	
Right orbita	Regular*	1.011	1.512	1.261±0.354	29.98
	Quick**	0.72	1.046	0.883±0.231	
Right ossification chain	Regular*	0.945	1.071	1.008±0.089	28.86
	Quick**	0.713	0.721	0.717±0.006	
Oral mucosa (Upper tongue, Hard palate)	Regular*	1.284	1.848	1.566±0.399	40.76
	Quick**	0.846	1.009	0.927±0.116	
C1-C2 (Spinal cord)	Regular*	1.13	1.293	1.212±0.115	46.52
	Quick**	0.619	0.677	0.648±0.041	
Right parotid	Regular*	1.491	1.495	1.493±0.003	38.63
	Quick**	0.903	0.929	0.916±0.018	
Left parotid	Regular*	1.027	1.39	1.209±0.251	39.22
	Quick**	0.687	0.783	0.735±0.068	
Sublingual gland	Regular*	1.281	1.516	1.399±0.166	43.67
	Quick**	0.743	0.832	0.788±0.063	
Right submandibular gland	Regular*	1.252	1.281	1.266±0.021	38.55
	Quick**	0.767	0.789	0.778±0.016	
Left submandibular gland	Regular*	1.291	1.509	1.400±0.154	46.98
	Quick**	0.721	0.764	0.742±0.031	
Thyroid (right)	Regular*	0.088	0.181	0.134±0.065	40.37
	Quick**	0.067	0.094	0.080±0.019	
Left lens	Regular*	1.377	1.526	1.452±0.106	45.36
	Quick**	0.739	0.847	0.793±0.077	
Thyroid skin	Regular*	0.394	0.465	0.43±0.05	62.88
	Quick**	0.114	0.205	0.159±0.065	
Effective Dose	Regular*	1.830			40.77
	Quick**	1.084			
Average Background Dose (Mean±SD)	-	0.077±0.004			-

Note: * Regular mode: 360^ 0^ rotation ** Quick mode: 180^ 0^ rotation.

**Table 5:** Minimum, maximum, mean, and standard deviations of the pixels in all VOIs (5x5 pixels) selected from paranasal sinus, maxilla, and mandible sites, representing bone, soft tissue, and air, in two imaging protocols, separately.

**Vertical Region**	**Imaging Protocol**	**Entity**	**VOI Number**	**Minimum**	**Maximum**	**Mean**±**SD**
Paranasal Sinus	Regular (360^ 0^)	Bone	1	1607	2331	2011.5±144.1
			2	894	2313	1940.9±182.7
			3	1633	2403	2087±150.7
		Soft tissue	4	-204	516	113.7±122.3
			5	-333	690	169.2±186.2
			6	-278	481	83.3±131.7
		Air	7	-770	-318	-563.3±74.9
			8	-829	-379	-613.5±58.5
			9	-855	-379	-636±91.6
	Quick (180^ 0^)	Bone	10	1237	2486	1828.7±188.4
			11	950	2042	1563.3±216.5
			12	1467	2513	2080.3±174.6
		Soft tissue	13	-227	543	108.9±118.1
			14	-493	826	142.5±233.2
			15	-275	361	55.6±113.1
		Air	16	-839	-223	-498.9±107.5
			17	-896	-350	-614.5±103.9
			18	-925	-188	-548.1±138.9
Maxilla	Regular (360^ 0^)	Bone	19	716	1852	1201.4±180.5
			20	723	1878	1281.9±181.1
			21	746	1646	1234.4±171.3
		Soft tissue	22	-365	455	84.4±127.5
			23	-269	310	24.7±104.3
			24	-433	141	-122.2±106.3
		Air	25	-1000	-933	-999.1±6.6
			26	-1000	-982	-999.9±1.4
			27	-989	-420	-773.7±97
	Quick (180^ 0^)	Bone	28	818	1603	1219.1±165.2
			29	534	1769	1298.3±156.5
			30	831	1832	1225.8±143.7
		Soft tissue	31	-283	449	55.9±128.3
			32	-385	414	33.4±130.2
			33	-559	127	-176.4±127.3
		Air	34	-1000	-925	-998.9±6.7
			35	-1000	-995	-1000.0±0.4
			36	-979	-413	-732.9±105.3
Mandible	Regular (360^ 0^)	Bone	37	995	2137	1566.7±253.3
			38	1442	2374	1952.1±209.8
			39	746	2150	1725.8±276.7
		Soft tissue	40	-309	98	-106±56.8
			41	-76	115	15.6±40.2
			42	-220	79	-67.4±48.6
		Air	43	-1000	-926	-998.9±6.1
			44	-1000	-994	-999.9±0.5
			45	-1000	-967	-999.6±2.5
Mandible
	Quick (180^ 0^)	Bone	46	715	1902	1500.6±262.6
			47	1417	2209	1888.4±152.9
			48	893	1950	1524.5±241.6
		Soft tissue	49	-330	83	-140.2±48.9
			50	-171	98	-11.4±40.7
			51	-208	7	-124.4±43.6
		Air	52	-1000	-975	-999.8±1.8
			53	-1000	-1000	-1000.0±0
			54	-1000	-971	-999.6±2.5

**Table 6:** Mean and standard deviation values of the bone, soft tissue, and air entities in the paranasal sinus, maxilla, and mandible sites were obtained by combining the average-pixel values calculated in three similar VOIs.

**Region**	**Tissue**	**Protocol**	**Minimum**	**Maximum**	**Mean**±**SD**
Paranasal Sinus	Bone	Regular*	1941	2087	2013.1±73.1
		Quick**	1563	2080	1824.1±258.5
	Soft tissue	Regular*	83	169	122.1±43.5
		Quick**	56	142	102.3±43.8
	Air	Regular*	-636	-563	-604.3±37.2
		Quick**	-615	-499	-553.8±58
Maxilla	Bone	Regular*	1201	1282	1239.2±40.5
		Quick**	1219	1298	1247.7±44
	Soft tissue	Regular*	-176	84	-4.4±106.3
		Quick**	-176	56	-29±128.1
	Air	Regular*	-1000	-774	-924.2±130.3
		Quick**	-1000	-733	-910.6±153.9
Mandible	Bone	Regular*	1567	1952	1748.2±193.7
		Quick**	1501	1888	1637.8±217.3
	Soft tissue	Regular*	-106	16	-52.6±62.1
		Quick**	-140	-11	-92±70.3
	Air	Regular*	-1000	-999	-999.5±0.5
		Quick**	-1000	-1000	-999.8±0.2

Note: * Regular mode: 360^ 0^ rotation ** Quick mode: 180^ 0^ rotation.

**Table 7:** Results of the SNR, CNR (or low-contrast resolution), and DIN methods demonstrate quantitative changes in image quality among two imaging protocols based on the axial slices selected from the paranasal sinus, maxilla, and mandible regions and the VOIs.

**Region**	**Quality Test**	**Regular Mode (360^ 0^)**	**Quick Mode (180^ 0^)**	**Quick/Regular Ratio**
Paranasal Sinus	SNR*	46.24	41.65	0.9
	CNR**	43.43	39.31	0.91
	DIN***	40.15	36.86	0.92
Maxilla	SNR*	11.66	9.74	0.84
	CNR**	11.7	9.97	0.85
	DIN***	10.56	8.66	0.82
Mandible	SNR*	28.14	23.31	0.83
	CNR**	28.99	24.62	0.85
	DIN***	26.41	21.46	0.81

Note: * SNR: Signal-to-noise ratio, ** CNR: Contrast-to-noise ratio, *** DIN: Deutsches Institut für Normung.

## Data Availability

All data generated or analyzed during this study are included in this published article.

## LIST OF ABBREVIATIONS

ART: Alderson radiation therapy
CT: Computed tomography
CBCT: Cone-beam computed tomography
CNR: Contrast-to-noise ratio
DICOM: Digital Imaging and Communications in Medicine
DIN: Deutsches Institut für Normung
ECCs: Element correction coefficients
EDs: Effective doses
EFOMP: European Federation of Organisations for Medical Physics
ESTRO: European Society for Therapeutic Radiation Oncology
FOV: Field of view
HU: Hounsfield unit
IAEA: International Atomic Energy Agency
ICRP: International Commission for Radiological Protection
ICRU: International Commission on Radiation Units and Measurements
kV: Kilovoltage
mA: Milliamperage
MRT: Morphology recognition technology
MSCT: Multi-slice computed tomography
MTF: Modulation transfer function
RCFs: Reader calibration factors
SID: Source-image receptor distance
SNR: Signal-to-noise ratio
TLDs: Thermoluminescent dosimeters
VOIs: Volume of interests
